# Roles of Hydration in Protein–Ligand Binding: Passive or Active Participant?

**DOI:** 10.1021/acs.jpca.5c04986

**Published:** 2025-09-30

**Authors:** Kacie A. Evans, He Mirabel Sun, Morgan Powers, Carter Lantz, Arthur Laganowsky, Hays Rye, David H. Russell

**Affiliations:** † Department of Chemistry, 14736 Texas A&M University, College Station, Texas 77843, United States; ‡ Department of Biochemistry and Biophysics, 14736 Texas A&M University, College Station, Texas 77843, United States

## Abstract

Hydration is a critical yet often underappreciated factor that influences protein dynamics in solution, with direct effects on structure, stability, and interactions such as ligand binding. Native mass spectrometry (nMS) enables the analysis of biomolecules in their solution states, which are shaped by cofactors, osmolytes, ligands, and notably, hydration. Here, we employ variable-temperature electrospray ionization to address a central question in molecular biophysics: does hydration act as a passive background solvent or as an active participant in modulating ligand binding? To investigate these effects, temperature-dependent changes in average charge state (*Z*_avg_), ADP equilibrium binding affinities (*K*_a_), and enthalpy–entropy compensation (EEC) for the GroEL single ring mutant (SR1) were collected in both H_2_O and D_2_O. Temperature-dependent shifts in *Z*_avg_ were observed for SR1-ADP complexes in both solvents, indicating protein conformational changes. Differences in nucleotide binding affinities calculated from mole fraction plots determined as a function of concentration between H_2_O and D_2_O solutions suggest that hydration plays a role in modulating ligand binding. Changes in hydration can modulate protein conformation and ligand binding affinities, typically reflected in shifts in enthalpy (Δ*H*) and entropy (−*T*Δ*S*), while the overall Gibbs free energy (Δ*G*) remains relatively unchanged. Thermodynamic analysis revealed distinct patterns of EEC in D_2_O compared to H_2_O, providing insight into how hydration modulates the SR1­(ADP)_1–7_ interactions. Collectively, these findings support the view that hydration acts as an active participant in ligand binding, with measurable effects on protein conformation, stability, and thermodynamics.

## Graphical abstract

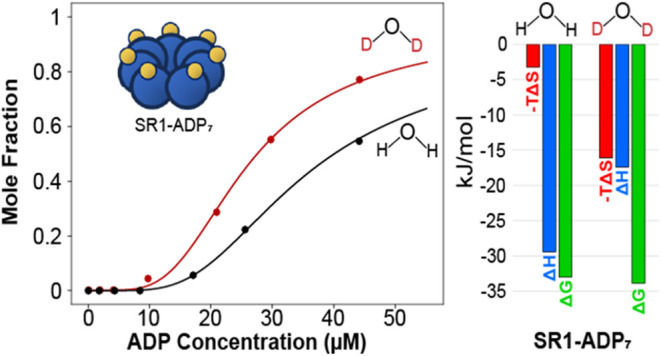

## Introduction

Proteins and protein complexes in solution populate ensembles of interconverting conformations that can be altered by changes in temperature, pH, ionic strength, and ligand binding.^1–3^ This conformational landscape is well described as an energy funnel, with a broad ensemble of unfolded conformations at the top of the funnel and the global free energy minimum, usually representing the native state, at the bottom.^4,5^ For many proteins, additional well-populated conformations also form in local energy minima between the unfolded ensemble and the native state. All of these macrostates (unfolded ensemble, intermediates, and native) are composed of collections of microstates that differ in various ways, e.g., the arrangement of the amino acid backbone and the degree of exposure of hydrophobic or polar groups.^6–8^ Protein folding also involves the interconversion of microstates that lie along the pathway to the formation of native states. Collectively, these microstates define a rugged energy landscape that shifts in response to changes in solution conditions.^5,9–11^ It is becoming increasingly recognized that changes in surface charge density can alter the charge state distribution observed by electrospray ionization mass spectrometry (ESI-MS), referred to here as the protonation microstate.^12^ Hydration,^13–20^ along with ionic strength,^21^ pH,^22^ ligands,^23,24^ and temperature,^25,26^ play central roles in dictating the dynamics and stability of protein complexes. Pan et al. recently showed that using a methanol–water mixture changes the conformational landscape of chymotrypsin inhibitor 2 (CI-2), underscoring how alterations in the solvent environment, and thus hydration, impact protein structure and dynamics.^27^ Similar effects have been observed with osmolytes, which can enhance or inhibit ligand binding depending on their influence on hydration.^28^ Together, these examples highlight hydration as a central determinant in protein dynamics. Understanding how hydration affects the distribution of protonation microstates can offer important insights into protein stability and binding mechanisms.

Dissecting shifts in microstate distributions influenced by changing solution conditions can be challenging, as each distribution consists of numerous states that can be “hidden” when using experimental approaches that report ensemble-averaged responses, e.g., isothermal titration calorimetry (ITC),^29^ X-ray diffraction (XRD),^14^ and cryogenic electron microscopy (cryo-EM).^30^ Previous studies have shown that mixed solvent systems, as mentioned above, are effective strategies for overcoming this limitation.^27^ In this context, water molecules themselves are known to exist in distinct dynamical subpopulations often described as “cold” (less dynamic) and “hot” (more dynamic), terms that refer to differences in molecular dynamics rather than thermal state.^20,31^ Cold water stabilizes protein structure, influencing both backbone and side chain interactions as well as ligand binding affinities.^13,17,32–35^ Temperature also contributes to the behavior of water, as water molecules interact more with hydrophobic regions at low temperatures, thereby altering protein conformation.^36–38^ Pressure can further modulate these unfolding pathways in ways that resemble cold unfolding.^39^ Because these subpopulations of water differ in dynamics, perturbing the solvent environment, such as by mixing H_2_O and D_2_O, can shift this balance. Integrating D_2_O alters the hydration sphere and enables the investigation of hydration-dependent changes in protein complexes.^40–46^ The physicochemical properties of H_2_O and D_2_O differ in terms of hydrogen bond strength, ionic strength, and temperature-dependent viscosity, which influences how water interacts with biomolecules.^43,47,48^ Most recently, D_2_O has been shown to increase the thermal stability of some protein complexes.^45,49,50^ Haidar and Konermann showed that these effects arise from solvent interactions rather than isotope effects.^51^ Moreover, changes in the hydration shell in D_2_O can influence ligand binding affinities and thermodynamics.^32,52^ Related work by Oliva et al. demonstrated that cosolvents such as DMSO can shift binding equilibria under pressure by altering hydration-dependent volumetric contributions.^53^ These findings support the idea that D_2_O can serve as a powerful probe for investigating the role of hydration in protein dynamics.

Mass spectrometry offers a unique advantage in this context due to its high mass resolution, which enables the detection of subtle hydration-dependent shifts in the intensities of protein–ligand complexes, particularly large ones.^54–57^ Ligand binding has previously been shown to be a sensitive indicator of shifts in the microstate distribution of proteins and protein complexes.^11^ For example, nMS combined with variable temperature (vT)-ESI has shown that ligand binding in the GroEL-ATP complex is highly temperature-dependent, implicating hydration effects.^23,58,59^ More recent studies have expanded this approach to examine how buffer composition modulates protein–ligand interactions.^6,59^ These findings support the broader utility of ligand binding as a tool for probing protein dynamics in varying environments, including hydration changes introduced by replacing H_2_O with D_2_O.^60,61^ One way to quantify these effects is through enthalpy–entropy compensation (EEC), which reports changes in enthalpy and entropy while Gibbs free energy remains constant. While EEC can result from multiple sources, including changes in hydration, protein conformation, and hydrolysis, careful system design can help isolate specific contributions. Here, we have selected the GroEL single ring mutant (SR1) to avoid effects such as GroEL negative inter-ring cooperativity and ATP hydrolysis.^62,63^ In this study, we apply vT-ESI-nMS to evaluate SR1-ADP binding in H_2_O and D_2_O. By comparing average charge state (*Z*_avg_), nucleotide equilibrium binding affinities (*K*_a_), and van’t Hoff plots with corresponding EEC analysis, our results collectively support the conclusion that the water network significantly impacts the stabilities and dynamics of SR1.

## Methods

### Sample Preparation

All chemicals including ammonium acetate (AmAc), ADP, and magnesium acetate (MgAc_2_) were purchased from Sigma-Aldrich (St. Louis, MO) and were dissolved in LC-MS grade deionized water. Each buffer had a final concentration of 200 mM after diluting from a 1 M stock solution using LC-MS grade deionized water. The corresponding 200 mM D_2_O buffers were made by diluting the 1 M stock in D_2_O purchased from Sigma-Aldrich (St. Louis, MO), resulting in an 80:20 ratio of D_2_O:H_2_O. All AmAc buffers (H_2_O and D_2_O) were adjusted to pH 7 using ammonium hydroxide. SR1 was overexpressed in *E. coli* as described previously.^64^ ADP sample aliquots containing 1 mM MgAc_2_ were stored at −20 °C and freshly diluted with the pH-adjusted 200 mM AmAc buffer containing 1 mM MgAc_2_, then added to the protein prior to analysis. Protein concentration was measured by using UV–vis at 280 nm. Fresh SR1 was diluted 3-fold and buffer exchanged into the corresponding buffer containing 1 mM MgAc_2_ by using Micro Bio spin P-6 gel column (Bio-Rad).

### Variable-Temperature Native Mass Spectrometry Analysis

The temperature of the solution contained in the nano-ESI emitter was controlled by the home-built variable temperature device as described previously.^58^ The vT-ESI temperature suggests an error of ± 1.5 °C. Solution temperatures used for this study were 4–50 °C, but the data for thermodynamics was limited to 5–35 °C. ADP solutions at various concentrations prepared in the same buffer as SR1 were titrated into SR1 and incubated at each temperature for 1 min. Then, mass spectra were collected on a Thermo Q Exactive UHMR (ultrahigh mass range) hybrid quadrupole orbitrap mass spectrometer. The resolution setting was maintained at 12500 with 5 microscans for SR1-ADP binding experiments. The capillary temperature was set to 120 °C with in-source trapping set to −200 V, and the HCD energy was set to 200. Using these conditions, no gas-phase dissociation products were observed. The acquisition time for each spectrum was set to 1 min.

### Data Processing

UniDec was used to assign the charge states, mass, and abundance of each individual species detected in the mass spectra.^65^ *Z*_avg_ was calculated as the weighted average of all charge states for a mass species. The integrated signal intensities of each complex were used to fit a sequential binding model for solving dissociation constant (*K*_d_) values as previously described by Cong et al.,^66^ from which the apparent binding constants (or the equilibrium constant *K*_eq_) are obtained as the reciprocals. The intrinsic binding constants (*K*_a_) are obtained using the equations reported from our past studies.^6,59^ The *K*_a_ values were used in the nonlinear van’t Hoff analysis to determine the thermodynamic profiles with the following equations from our past studies.^6^ The Gibbs free energy for ADP binding can be calculated by using eq 1. Enthalpy and the change in heat capacity for the binding reaction at the temperature T_0_ were derived using the nonlinear van’t Hoff equation^67^ (eq 2), which includes a temperature-dependent heat capacity value.

1 $$\Delta G=-\mathrm{RT}\ln K_{a}$$

2 $$\ln K_{a}=\ln K_{0}+\frac{{\Delta C}_{p}}{R}•\ln\frac{T}{T_{0}}+\left(\frac{{\Delta H}_{0}-T_{o}•{\Delta C}_{p}}{R}\right)•\left(\frac{1}{T_{0}}-\frac{1}{T}\right)$$

*R = 8.314 J·K^–1^·mol^–1^, *K*_0_ is the intrinsic binding constant at *T*_0_. The magnitude of Δ*S* at *T*_0_ can then be calculated from the Δ*H*_0_ and Δ*G*_0_ values using eq 3.

3 ΔG=ΔH−TΔS

## Results

Replacing H_2_O with D_2_O alters several key properties of the SR1-ADP complex, including its temperature-dependent *Z*_avg_, ligand binding affinities, and thermodynamic profiles. All experiments were conducted after the sample was incubated for 48 h at 4 °C, when hydrogen–deuterium exchange reached equilibrium at approximately 46–48% (see Figures S1 and S2). Using vT-ESI, *Z*_avg_ of SR1 and SR1­(ADP)_1–7_ complexes were monitored over a range of temperatures (5–50 °C) in both H_2_O and D_2_O. ADP binding and the associated thermodynamics were observed to differ in D_2_O and H_2_O. Collectively, the data presented across all experiments demonstrate that changes in hydration in D_2_O significantly impact stabilities and dynamics of the SR1 complex.

VT-ESI experiments were conducted to assess how temperature influences SR1 conformational dynamics and stability in 80% D_2_O compared to H_2_O. Figure 1 contains plots showing temperature-dependent (5–50 °C) *Z*_avg_ changes for SR1 and SR1­(ADP)*_n_* (*n* = 1–7) in H_2_O and 80% D_2_O. Solution phase thermal dissociation of SR1 is observed for both solutions at 45 °C (see Figure S3), limiting this study to 50 °C. Note that fewer thermal dissociation products are observed at 45 °C in D_2_O in relation to H_2_O, consistent with increased thermal stability previously reported in D_2_O.^45,46,50^ *Z*_avg_ serves as an indicator of changes in the solvent accessible surface area (SASA), which reflects temperature-induced conformational shifts.^68,69^ From 5–20 °C, a slight decrease in *Z*_avg_ was observed for the SR1 and SR1­(ADP)*_n_* complexes in both H_2_O and 80% D_2_O, with D_2_O showing a slightly greater decrease in *Z*_avg_ of ∼0.5 at 20 °C. Above 20 °C, *Z*_avg_ increased significantly in both solvents. A minimum in *Z*_avg_ at 20 °C reflects a transition point: below this temperature, cold conformational changes dominate, driven by hydration water restructuring and reduced flexibility of the protein complex, whereas above it, hot conformational changes occur, reflecting thermally activated rearrangements and increased conformational dynamics.^34,37,70^ Evaluating the effect of temperature on SR1 and SR1-ADP complexes indicates that in 80% D_2_O, the stability of the complex is different, despite similar overall conformational trends.

**Figure 1:**
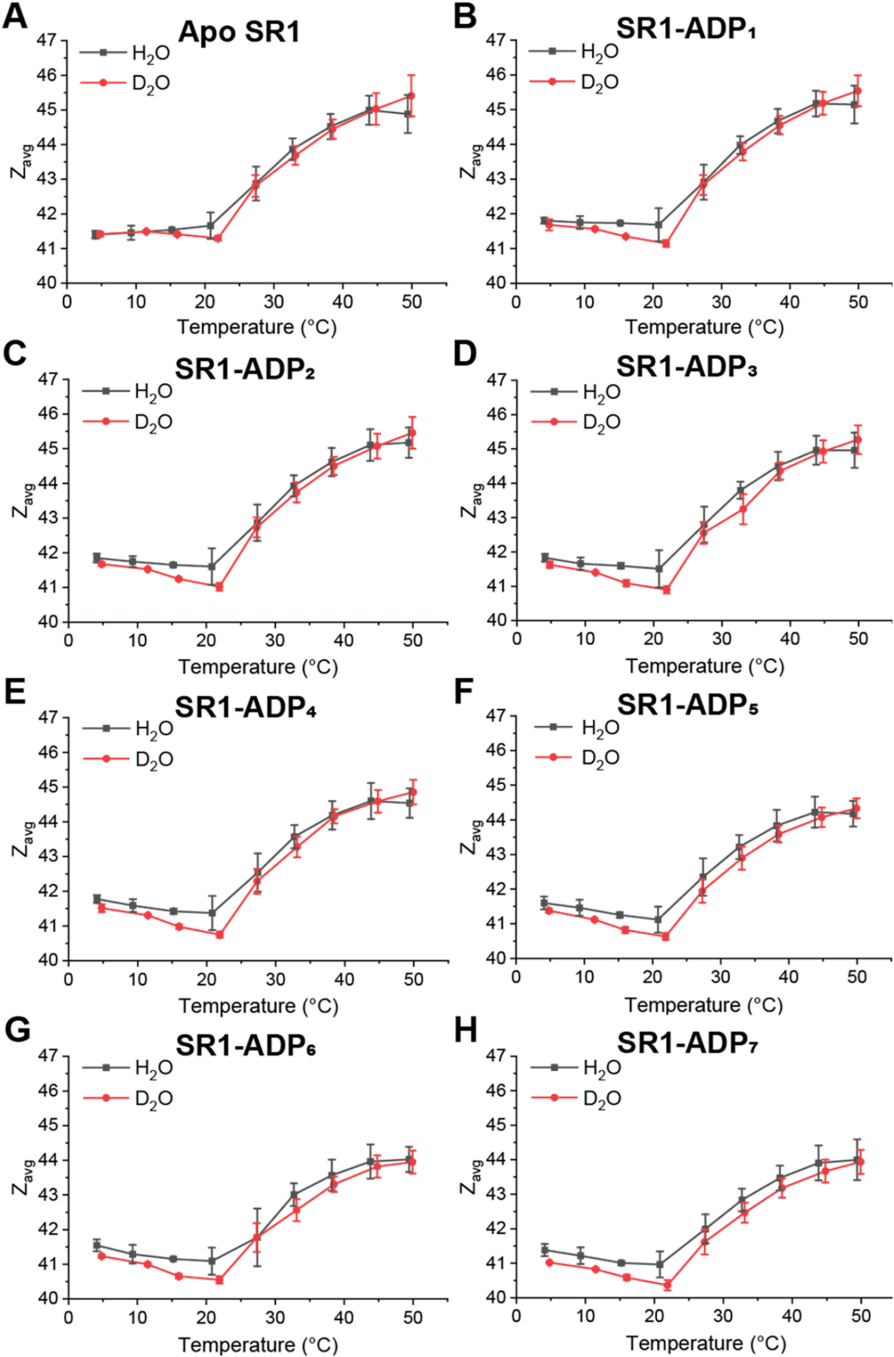
Effects of solution temperature contained in the ESI emitter on the *Z*_avg_ for SR1 (A) and SR1­(ADP)*_n_* complexes (B–H) in H_2_O with 25 μM ADP (black) and in 80% D_2_O with 15 μM ADP (red). The averaged data represent triplicate measurements, and error bars represent standard deviation (*n* = 3).

The binding affinities of the SR1­(ADP)*_n_* (*n* = 1–7) complexes were determined to evaluate how ADP binding is altered for the SR1 complex in the presence of D_2_O compared to H_2_O. Deconvoluted mass spectra for SR1-ADP binding in H_2_O and 80% D_2_O acquired at 5, 20, and 50 μM ADP concentrations are shown in Figure 2A. Mass assignment data are contained in Tables S1 and S2. For each ADP concentration shown, more extensive ADP binding was observed in 80% D_2_O compared to H_2_O. Mole fraction plots at 25 °C shown in Figure 2B, show that the SR1­(ADP)_7_ complex reaches 50% mole fraction at ∼25 μM ADP in 80% D_2_O, compared to ∼35 μM in H_2_O. At 50 μM ADP, SR1­(ADP)_7_ reaches ∼80% mole fraction in 80% D_2_O, but only ∼60% in H_2_O. Equilibrium binding constants (*K*_a_) calculated from the relative intensities of individual mass species, as previously reported by Cong et al.^66^ and are shown in Figure 2C. Binding constants have been statistically corrected to account for the number of binding sites (seven in this case), consistent with our previous studies.^6,59^ The *K*_a_ for the seventh binding is approximately twice as high in 80% D_2_O compared to H_2_O, while the first six ADP bindings show a less significant increase. The increased binding affinity for the final binding step is consistent with reports of positive cooperativity in GroEL and SR1, where nucleotide binding stabilizes the transition from a T to an R-like state.^62,71^ These results indicate that D_2_O changes the ligand binding mechanism, particularly for the final ADP binding, suggesting altered hydration near the binding site, prompting further thermodynamic analysis.

**Figure 2:**
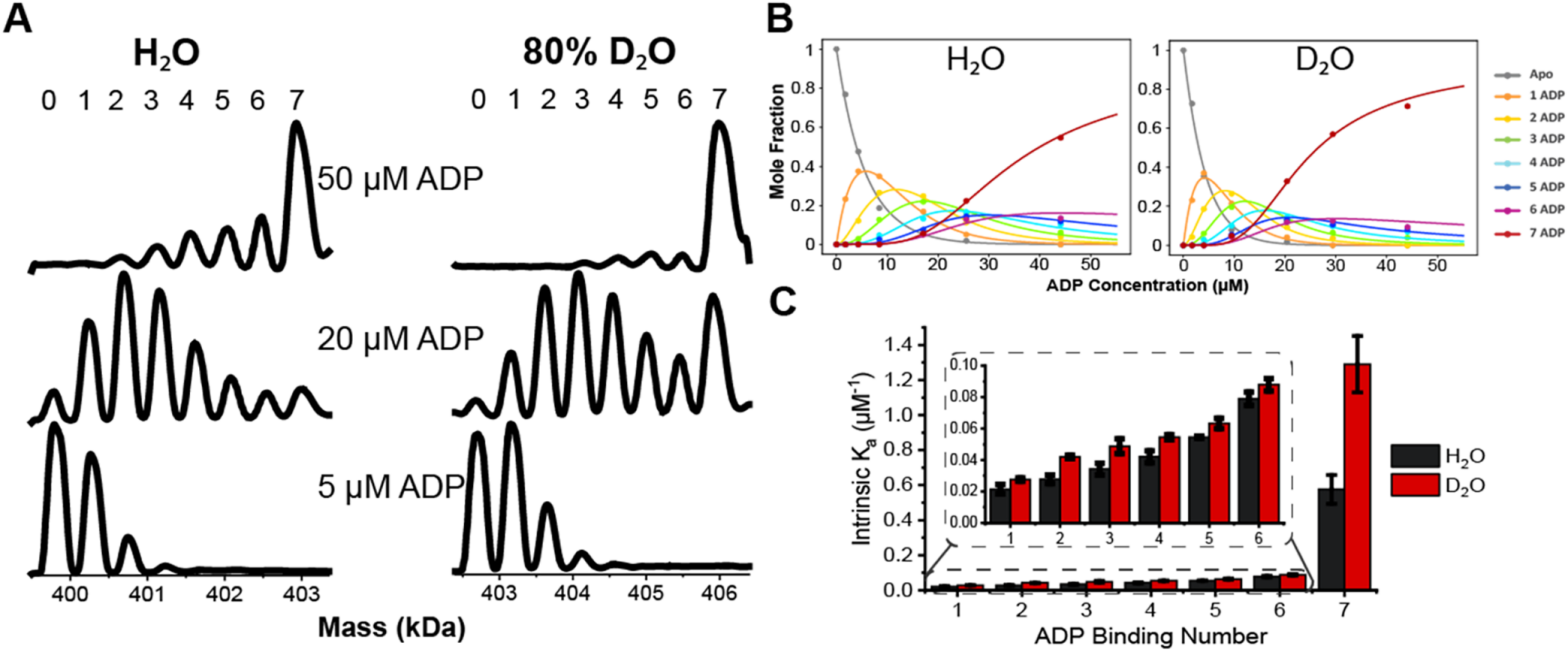
(A) Deconvoluted mass of SR1 in H_2_O and 80% D_2_O with varying concentrations of ADP. (B) Corresponding mole fraction plots for SR1­(ADP)*_n_* (*n* = 0–7) complexes in H_2_O and 80% D_2_O at 25 °C. (C) Bar charts showing intrinsic binding constants (*K*_a_) for individual SR1-ADP binding steps in H_2_O (black) and 80% D_2_O (red). All binding constants are generated from triplicated data sets taken at 25 °C, and error bars represent standard deviation (*n* = 3).

Van’t Hoff plots shown in Figure 3A,B were generated from the data shown in Figure 2 to determine the thermodynamic effects for each ADP binding to SR1. The nonlinear behavior displayed indicates a temperature-dependent change in heat capacity (Δ*C*_p_), which may arise from water reorganization, protein conformational shifts, or desolvation during ligand binding.^72^ An inflection point at ∼21 °C is observed in each van’t Hoff plot, aligning with the *Z*_avg_ minima shown in Figure 1. In H_2_O, the first five ADP bindings display convex van’t Hoff curves, reflecting positive Δ*C*_p_. The sixth and seventh bindings show more linear behavior. In 80% D_2_O, the first six bindings are similar to those in H_2_O, but the seventh binding is less linear, suggesting different hydration effects. The curvature of the van’t Hoff plots is representative of the Δ*C*_p_ values, which are reported in Table S3. The differences in curvature between H_2_O and D_2_O highlight altered thermodynamic responses, which are most likely tied to changes in the hydration sphere around the surface of SR1.

**Figure 3:**
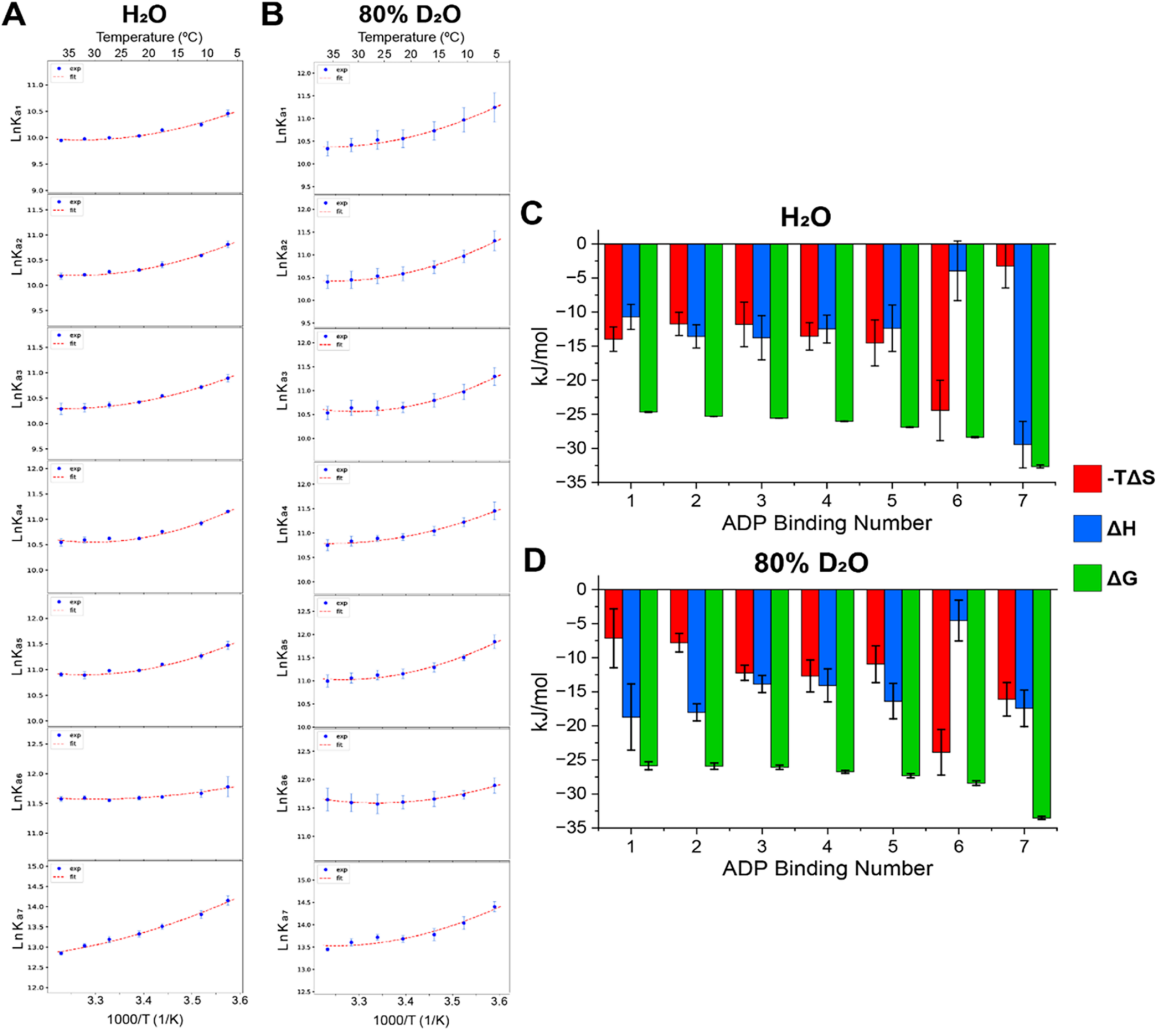
(A) The van’t Hoff plots are shown in H_2_O and (B) 80% D_2_O for SR1­(ADP)*_n_* (*n* = 1–7) complexes. The corresponding entropy, enthalpy, and free energy values for individual ADP binding steps in (C) and (D), respectively, for individual ADP binding steps at 25 °C are shown in bar charts. All values are generated from triplicated data sets, and error bars represent standard deviation (*n* = 3).

Thermodynamic profiles (Δ*G*, Δ*H*, and −*T*Δ*S*) at 25 °C for each SR1-ADP binding reaction in H_2_O and 80% D_2_O are shown in Figures 3C,D, respectively. While free energy remains relatively constant, significant shifts in enthalpy and entropy indicate EEC, consistent with prior studies on GroEL-ATP and SR1-ADP binding.^6,23,59^ In 80% D_2_O, the first and second ADP bindings are more entropy-driven, whereas in H_2_O, both enthalpy and entropy contribute comparably. The third–fifth bindings show similar thermodynamic profiles in both H_2_O and 80% D_2_O. The sixth binding is predominantly entropy-driven in both H_2_O and 80% D_2_O. Interestingly, the seventh binding has almost equivalent enthalpy–entropy contributions in 80% D_2_O but is enthalpy-driven in H_2_O. The shifts in thermodynamic profiles between H_2_O and 80% D_2_O strongly suggest that altered hydration impacts the ligand binding mechanism. Overall, these results demonstrate that D_2_O-induced changes in hydration affect multiple aspects of the SR1 complex, including stabilities, ligand-binding affinities, and thermodynamic profiles.

## Discussion

The use of 80% D_2_O in place of H_2_O alters key properties of the SR1 complex, including stabilities, ADP binding affinities, and thermodynamic profiles. These differences highlight how changes to the hydration sphere influence the ligand binding mechanism of SR1. By employing ligand binding as a probe, we show that D_2_O-induced alterations in the water network contribute to the modulation of protein–ligand interactions. The combined results from the *Z*_avg_ analysis, binding affinities, and thermodynamic profiles emphasize that changes in hydration affect the conformational dynamics of SR1, as well as the energetics of its interaction with ADP.

Temperature-dependent shifts in *Z*_avg_ shown in Figure 1 suggest that hydration contributes to alterations in the SASA and thermal stability of SR1. *Z*_avg_ shifts reflect changes in SASA due to the restructuring of protein subunits, where a decrease indicates buried residues and an increase reflects greater solvent exposure.^68,69^ Below 20 °C, a decrease in *Z*_avg_ in both H_2_O and D_2_O suggests cold-induced restructuring of the complex, whereas the increase in *Z*_avg_ above 20 °C reflects hot conformation changes. These transitions align with the two-state model for water structure and its known temperature-dependent effects on protein stability.^34,37,73–76^ While overall *Z*_avg_ values are similar in both H_2_O and D_2_O, the minimum in D_2_O is lower compared to H_2_O at 20 °C, which marks the inflection point where protein conformational restructuring behavior changes.^34,36^ This difference in *Z*_avg_ may reflect altered restructuring of the water network in D_2_O compared to that in H_2_O at this inflection point. Additionally, reduced thermal dissociation products observed in D_2_O at 45 °C are consistent with prior reports that protein thermal stability was increased in D_2_O.^45,46,50^ The lack of large changes in *Z*_avg_ between H_2_O and D_2_O further supports the conclusion that D_2_O increases SR1 stability without inducing major conformational shifts.

ADP binding affinities shown in Figure 2 are increased in D_2_O, particularly for the seventh ADP binding, which shows a 2-fold increase in *K*_a_ compared to H_2_O. The alteration of ligand binding can be attributed to the protein dynamics, hydrogen bond networks, solvation of proteins and ligands, as well as the displacement of water molecules in the binding cavity. Among these factors, the observed difference in binding affinity in D_2_O and H_2_O can potentially be attributed to changes in the binding site water network.^52^ The shift in EEC observed in the thermodynamic profiles shown in Figure 3 further supports this interpretation. For the first and second ADP bindings, D_2_O leads to increased enthalpy and decreased entropy compared to H_2_O. This shift may result from increased rigidity of the protein complex in D_2_O due to stronger solvent–solvent hydrogen bonding. Increased rigidity has been previously reported for protein complexes in D_2_O.^41,42^ The stronger solvent–solvent interactions in D_2_O reduce the flexibility of the hydration shell, which in turn decreases the entropy that corresponds with reorganization of water molecules upon ligand binding. In contrast, the seventh ADP binding displays decreased enthalpy and increased entropy in D_2_O compared to H_2_O. The increased entropy observed can be interpreted as the displacement of water molecules from the binding pocket that aligns with the structural extension of the GroEL subunits as the nucleotide ligand binds.^77–82^ The greater entropy observed for the seventh binding in D_2_O suggests continued rearrangement of hydration, whereas in H_2_O, the reorganization appears largely complete. These shifts in EEC reinforce the idea that cold, structured water near the binding pocket plays an important role in SR1 stability and the ligand binding mechanism.

Nonlinear van’t Hoff plots shown in Figure 3 (A and B) provide insights into solvent reorganization and conformational changes accompanying ligand binding. The curvatures observed in each plot are associated with a positive Δ*C*_p_ value, which is typically ascribed to desolvation, water reorganization, and protein structural transitions.^83–85^ Most of the binding steps show comparable nonlinearity in both H_2_O and D_2_O, suggesting similar changes in hydration upon nucleotide binding. However, the seventh ADP binding displays greater linearity in H_2_O than in D_2_O, implying smaller Δ*C*_p_ and fewer solvent or conformational changes during this final binding step in H_2_O. This interpretation aligns with the lower entropy change observed in the EEC profile for SR1­(ADP)_7_, in H_2_O, and supports the idea that rearrangement of water owing to the protein conformational change associated with the final ADP binding is more complete in H_2_O by the seventh binding. In D_2_O, the sustained curvature of the van’t Hoff plot and higher entropy contribution imply continued restructuring of the hydration shell with the seventh binding. Together, these findings emphasize the role of hydration in modulating the ligand binding mechanism for SR1.

## Conclusion

The results presented here highlight the active role of hydration in modulating the stabilities and dynamics of SR1, as evidenced by differences observed in D_2_O versus H_2_O solutions. The stabilizing effects of D_2_O have previously been shown to arise from changes in solvent interactions rather than isotope effects, allowing the observed thermodynamic differences to be attributed to altered hydration.^51^ Distinct physicochemical properties of H_2_O and D_2_O prompt changes in solvent–solvent and solvent–protein interactions, which in turn influence ligand binding. Although *Z*_avg_ values are similar between H_2_O and D_2_O, a consistent decrease of *Z*_avg_ at 20 °C in D_2_O corresponds to the temperature where water transitions from cold to hot restructuing,^36,76^ consistent with altered water structuring around both the protein subunits and the ligand binding site. Collectively, the changes observed for temperature-dependent *Z*_avg_, ADP binding affinities, and thermodynamic measurements further suggest that protein dynamics are enslaved to hydration.^14^ While increased ADP binding affinity in D_2_O demonstrates the influence of hydration, the complementary shifts in EEC provide a more nuanced and complete picture. One speculative interpretation of the increased entropy contribution seen for the seventh ADP binding in D_2_O is that the SR1 binding cavity may adopt a slightly expanded conformation in D_2_O, facilitating the rapid influx or rearrangement of water molecules during the final ligand binding reaction. The rapid influx of water into an enlarged cavity may contribute to the increased entropy observed for the seventh ADP binding, as has been reported in rhodopsin, where cavity expansion facilitates water influx and generates entropic contributions during activation.^77,86,87^ This study demonstrates that thermodynamic analysis adds a critical dimension to the characterization of hydration effects by enabling the dissection of EEC for ligand binding on a per-binding basis. Overall, these findings support the view that hydration is not a passive background solvent but an active participant that influences the conformational stability and ligand binding mechanisms of protein complexes.

## Supplementary Material



## Notes

Intact hydrogen–deuterium exchange of SR1, mass spectra of SR1 in H_2_O and D_2_O after 48 h, mass spectra of SR1 thermal denaturation in H_2_O and D_2_O, mass assignment information on SR1­(ADP)*_n_* complexes in H_2_O and D_2_O, and heat capacity changes (PDF)
