# Supplementary material for: Roles of Hydration in Protein–Ligand Binding: Passive or Active Participant?
Source: J Phys Chem A. 2025 Sep 30;129(40):9477–85. doi: 10.1021/acs.jpca.5c04986 (PMC12516730; doi:10.1021/acs.jpca.5c04986)
Supplement: Supplementary file 1 [file jp5c04986_si_001.pdf]

Supporting Information

Roles of Hydration in Protein-Ligand Binding: Passive or Active Participant?

Kacie A. Evans,[†] He Mirabel Sun,[†] Morgan Powers,[‡] Carter Lantz,[†] Arthur Laganowsky,[†] Hays Rye[*,‡] and David H. Russell[*,†]

[†]Department of Chemistry, Texas A&M University, College Station, Texas 77842

[‡]Department of Biochemistry and Biophysics, Texas A&M University, College Station, Texas 77842

*Email: russell@chem.tamu.edu.
*Email: hays.rye@ag.tamu.edu.

Table of Contents

	Page
**Figure S1.** Intact hydrogen deuterium exchange of SR1 over time	S2
**Figure S2.** Mass Spectrum of SR1 in H_2O and D_2O	S2
**Figure S3.** Mass spectrum comparing thermal dissociation between H_2O and D_2O	S3
**Table S1.** Deconvoluted mass of SR1 and SR1-ADP$_n$ in H_2O and D_2O	S3
**Table S2.** Full width half maximum of the deconvoluted MS peaks of SR1 and SR1-ADP$_n$ complexes in H_2O and D_2O	S4
**Table S3.** Values of heat capacity change (ΔCp, $kJ \cdot mol^{-1} \cdot K^{-1}$) for individual binding steps in H_2O and D_2O	S4

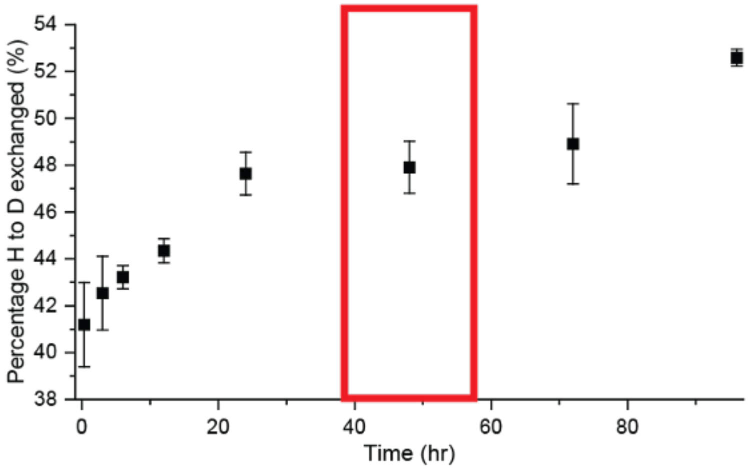

Figure S1. SR1 was diluted in 80% D_2O, and the percentage of exchangeable hydrogen exchanged with deuterium is plotted. The red box highlights the time frame at which SR1-ADP experiments occurred in D_2O for this study.

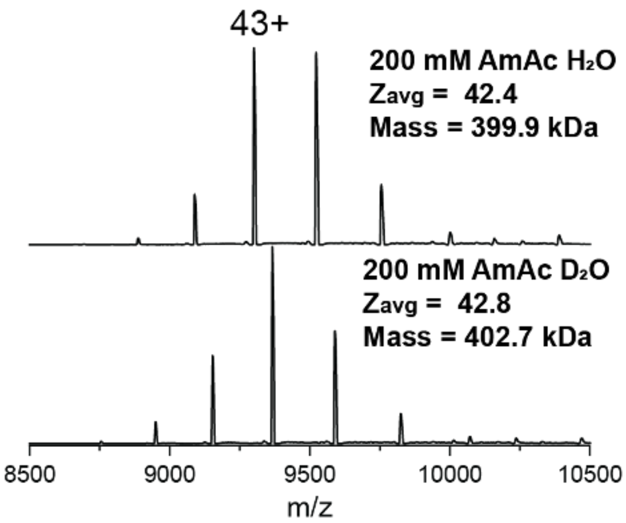

Figure S2. Mass spectra with mass and Z_{avg} values for SR1 in H_2O and 80% D_2O after being incubated at 4 °C for 48 h.

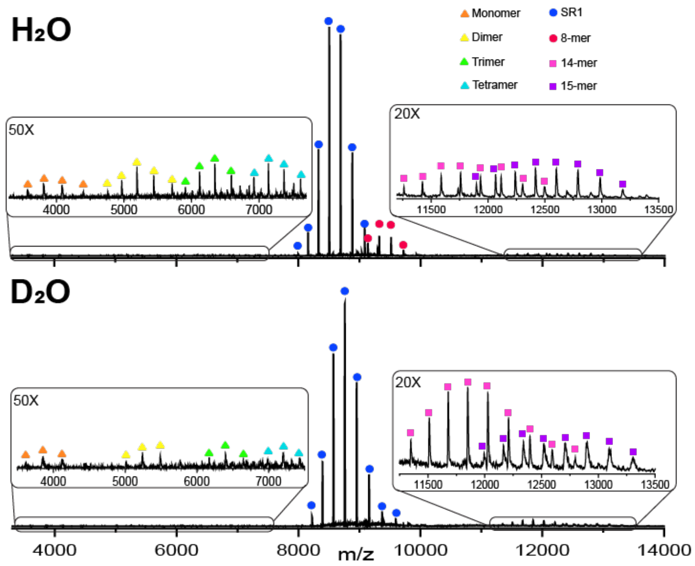

Figure S3. The mass spectra in H_2O and 80% D_2O at 45 °C, showing the thermal dissociation products for the SR1 complex.

Table S1. Deconvoluted mass (kDa) of SR1-ADP$_n$ in H_2O and 80% D_2O after 48 h of incubation at 4 °C. ΔMass represents the change in mass of each ADP addition in relation to the reported Apo SR1 mass. The values are the averages of triplicated data sets.

	H_2O			80% D_2O		
	Mass (kDa)	Mass Standard Deviation (Da)	ΔMass (Da)	Mass (kDa)	Mass Standard Deviation (Da)	ΔMass (Da)
SR1 (apo)	399.78	18		402.68	22	
SR1-ADP$_1$	400.24	15	458	403.17	19	489
SR1-ADP$_2$	400.69	13	907	403.64	23	959
SR1-ADP$_3$	401.14	13	1,362	404.10	28	1,422
SR1-ADP$_4$	401.60	10	1,816	404.57	25	1,885
SR1-ADP$_5$	402.05	16	2,273	405.02	29	2,342
SR1-ADP$_6$	402.52	3	2,740	405.47	22	2,788
SR1-ADP$_7$	402.96	17	3,182	405.92	24	3,242

Table S2. Full width at half maximum of each peak for the deconvoluted mass spectra of SR1-ADP_n in H_2O and 80% D_2O (kDa).

	H_2O	80% D_2O
SR1 (apo)	0.25	0.28
SR1-ADP_1	0.21	0.22
SR1-ADP_2	0.23	0.23
SR1-ADP_3	0.22	0.24
SR1-ADP_4	0.22	0.23
SR1-ADP_5	0.22	0.23
SR1-ADP_6	0.21	0.22
SR1-ADP_7	0.26	0.30

Table S3. Values of heat capacity change (ΔC_p, unit in kJ·mol-1·K-1) in H_2O and 80% D_2O for individual ADP binding steps, respectively. All values are obtained through van't Hoff analysis shown in the main text (**Figure 3**), generated from triplicated data sets.

	H_2O	R^2	80% D_2O	R^2
SR1-ADP_1	0.99±0.35	0.98	1.24±0.41	0.99
SR1-ADP_2	1.14±0.33	0.99	1.42±0.65	0.99
SR1-ADP_3	0.98±0.65	1.00	1.52±0.57	0.97
SR1-ADP_4	1.45±0.55	0.97	0.99±0.51	0.99
SR1-ADP_5	1.22±0.42	0.98	1.43±0.13	0.98
SR1-ADP_6	0.48±0.57	0.95	0.9±0.35	0.99
SR1-ADP_7	0.91±0.15	0.99	1.32±0.28	0.95
